# Estimated Change in Prevalence of Hypertension in Nepal Following Application of the 2017 ACC/AHA Guideline

**DOI:** 10.1001/jamanetworkopen.2018.0606

**Published:** 2018-07-13

**Authors:** Gulam Muhammed Al Kibria, Krystal Swasey, Angela KC, Mohammadhassan Mirbolouk, Muhammad Nazmus Sakib, Atia Sharmeen, Mahmuda Jahan Chadni, Kristen A. Stafford

**Affiliations:** 1 Department of Epidemiology and Public Health, School of Medicine, University of Maryland, Baltimore; 2 Bloomberg School of Public Health, Johns Hopkins University, Baltimore, Maryland; 3 Johns Hopkins Hospital, Baltimore, Maryland; 4 Impulse Hospital, Tejgaon, Dhaka, Bangladesh; 5 School of Community Health and Policy, Morgan State University, Baltimore, Maryland; 6 Nuffield Department of Population Health, University of Oxford, Oxford, United Kingdom

## Abstract

**Importance:**

The 2017 American College of Cardiology/American Heart Association (ACC/AHA) Guideline for the Prevention, Detection, Evaluation, and Management of High Blood Pressure in Adults lowered the systolic and diastolic blood pressure thresholds for hypertension to 130 and 80 mm Hg, respectively. This represents a reduction of 10 mm Hg in both systolic and diastolic blood pressure levels used to define hypertension compared with previous guidelines, such as the Seventh Report of the Joint National Committee on Prevention, Detection, Evaluation, and Treatment of High Blood Pressure (JNC 7).

**Objectives:**

To estimate the prevalence of hypertension among adults aged 18 years or older in Nepal per the 2017 ACC/AHA guideline and to determine the absolute difference in hypertension prevalence comparing the 2017 ACC/AHA and JNC 7 guidelines.

**Design, Setting, and Participants:**

The cross-sectional analysis used data from the population-based 2016 Nepal Demographic and Health Survey. Data were collected from June 2016 to January 2017 using a multistage stratified sampling procedure that was applied in urban and rural areas, using wards as the primary sampling units. Individuals aged 15 years or older from selected households were interviewed. The survey had an overall response rate of approximately 97%.

**Main Outcomes and Measures:**

The primary outcome was the prevalence of hypertension. Blood pressure was measured 3 times for each participant with 5-minute intervals between. Hypertension was present if blood pressure was greater than or equal to 130/80 mm Hg for the 2017 ACC/AHA guideline, and greater than or equal to 140/90 mm Hg for the JNC 7 guideline.

**Results:**

Among 13 519 participants (median [interquartile range] age, 38 [26-53] years; 7821 [57.9%] female), 44.2% (95% CI, 43.4%-45.0%; n = 5977) had hypertension according to the 2017 ACC/AHA guideline compared with 21.2% (95% CI, 20.5%-21.9%; n = 2869) by the JNC 7 guideline. The new prevalence was associated with an absolute increase of 23.0% (95% CI, 22.3%-23.7%) from the JNC 7 guideline. When estimating the proportion of hypertension by background characteristics, the new 2017 ACC/AHA guideline definition increased the prevalence to 50% or greater for some categories, with the highest prevalence among those with a body mass index (calculated as weight in kilograms divided by height in meters squared) greater than or equal to 30 (71.6%; 95% CI, 67.7%-75.3%) and between 25 and 29.9 (62.1%; 95% CI, 60.1%-64.1%).

**Conclusions and Relevance:**

For adults in Nepal, the new 2017 ACC/AHA guideline reveals a greater estimated prevalence of hypertension compared with the JNC 7 guideline. Because of the public health significance of hypertension, higher prevalence rates confirm the importance of developing effective prevention and control methods in this country.

## Key Points

**Question:**

What is the change in prevalence of hypertension among adults in Nepal due to lowering the blood pressure threshold in the 2017 American College of Cardiology/American Heart Association (ACC/AHA) hypertension guideline?

**Findings:**

Using data from the nationally representative 2016 Nepal Demographic and Health Survey, the estimated prevalence of hypertension was 44.2% according to the 2017 ACC/AHA guideline, 23% higher than the estimate under previous guidelines (21.2%).

**Meaning:**

The new estimates suggest that nearly half of adults in Nepal may have hypertension; these results support the need for public health programs to increase awareness and minimize complications of hypertension.

## Teaser

This cross-sectional analysis uses data from a population-based survey of residents of Nepal to identify changes in the prevalence of hypertension according to the 2017 American College of Cardiology/American Heart Association (ACC/AHA) hypertension guideline compared with previous guidelines.

## Introduction

The 2017 American College of Cardiology/American Heart Association (ACC/AHA) Guideline for the Prevention, Detection, Evaluation, and Management of High Blood Pressure in Adults lowered the threshold for the definition of hypertension. The 2017 ACC/AHA guideline describes the condition as a systolic blood pressure (SBP) greater than or equal to 130 mm Hg or a diastolic blood pressure (DBP) greater than or equal to 80 mm Hg.^1^ Previous guidelines, such as the 1999 World Health Organization–International Society of Hypertension Guideline (1999 WHO-ISH)^2^ and the Seventh Report of the Joint National Committee on Prevention, Detection, Evaluation, and Treatment of High Blood Pressure (JNC 7), defined hypertension as an SBP greater than or equal to 140 mm Hg or a DBP greater than or equal to 90 mm Hg.^3^ Application of this revised definition may reclassify a significant proportion of people as hypertensive who were previously categorized as prehypertensive, or people with high normal blood pressure. The benefit of this would be to potentially catch individuals earlier in disease progression and reduce cardiovascular morbidity and mortality.^1^ This new evidence-based recommendation has prompted researchers to estimate an updated prevalence of hypertension; a growing body of literature has recognized the importance of the adjusted definition.^4,5,6^ Implications of this new guideline highlight the importance of having revised estimates for all countries to aid public health resource planning and prevention strategies, as the prevalence of hypertension varies by region. The estimates that used the previous definition of 140/90 mm Hg found that prevalence of hypertension could vary according to age and sex, as well as education and other sociodemographic characteristics.^1,6^ While prevalence is not the ideal measure by which to estimate risk factors for a clinical condition, it may be useful to identify subgroups who may benefit from public health interventions.

A number of studies to date have estimated the adjusted prevalence of hypertension in some countries according to the new guideline.^5,6^ Muntner et al^6^ examined the effects of the new definition by assessing the hypertension prevalence in the United States and found an absolute increase of 14.7% among people aged 20 years or older. Additionally, Khera et al^5^ estimated the relative increase of prevalence among adults aged 45 to 75 years in the United States and China and found an overall relative increase of 45.1% and 26.8% in these 2 countries, respectively. Estimating an updated prevalence for developing countries could be of particular interest because there is not only a lack of information available from these regions, but also an increasing burden of cardiovascular diseases.^7,8,9,10^ Although prior studies have shown that hypertension and other cardiovascular diseases were the leading causes of global deaths and disability-adjusted life-years,^7,8,9^ updated measures may more accurately demonstrate the burden of this illness.

The 2016 Nepal Demographic and Health Survey (2016 NDHS) included blood pressure measurements among the variables collected from the study sample.^10^ Nepal is a developing country in South Asia with an estimated land mass of 143 351 km^2^ and a population of about 29 million. This landlocked country is divided into 7 administrative provinces.^11^ Most of the population is engaged in agriculture, and nearly 59% of people reside in urban regions.^10,11^ Using the 1999 WHO-ISH guideline, among people aged 15 years or older, the 2016 NDHS estimated the hypertension prevalence as 23% and 17% among men and women, respectively.^10^

The aim of this study was to estimate the prevalence of hypertension among adults (aged ≥18 years) in Nepal after applying the new 2017 ACC/AHA guideline. In addition, we compared this new 2017 ACC/AHA prevalence with the prevalence from the older JNC 7 guideline’s hypertension definition to determine the absolute differences in the prevalence of this condition according to stages and background characteristics.

## Methods

### Data Source

This study analyzed the cross-sectional survey data set of the 2016 NDHS. The survey was implemented from June 2016 to January 2017. New ERA, a private research organization in Nepal, conducted this survey. The main objective of this survey was to provide updated estimates of demographic and health indicators. The consulting services company ICF provided technical assistance to the survey as well as approval to use the data for secondary analyses. The Nepal Health Research Council and the ICF institutional review board approved the 2016 NDHS survey protocol. The head of household provided written informed consent before the interview.^10^ We obtained the approval to use the data for the current study from ICF in January 2018. The institutional review board of the University of Maryland, Baltimore, exempted the study from oversight as it was not human subjects research.

### Study Population and Survey Design

An updated version of the frame from the 2011 Population and Housing Census was used in the sampling frame. A 2-stage stratified sample was used in rural areas. The primary sampling units in rural areas were wards (n = 199). The households were selected from the primary sampling units in the second stage.^10^

In urban regions, the sample was selected in 3 stages. The primary sampling unit was a ward (n = 184). Then, 1 enumeration area was randomly selected from each ward, and households were selected from enumeration area. Each cluster (enumeration area or ward) expected to have 30 households, which would yield a total of 11 490 households. All women and men aged 15 years or older were eligible for blood pressure measurements in half of the households.^10^

These households were then visited and interviewed. The overall response rate was approximately 97%. A total unweighted sample of 5571 men and 7861 women aged 18 years or older was interviewed. Details of this population-based survey, including survey design, methods, questionnaires, and sample size determination, have been described elsewhere.^10^

### Measurements

The blood pressure of the survey participants was measured with UA-767F/FAC blood pressure monitors (A&D Medical). Blood pressure was measured 3 times for each individual with an interval of 5 minutes between the measurements. The mean of the last 2 measurements was used to define and categorize the final pressure level.^10^

### Definition of Hypertension

According to the JNC 7 guideline, individuals who have an SBP greater than or equal to 140 mm Hg or a DBP greater than or equal to 90 mm Hg or take any prescribed drugs to control blood pressure were categorized as hypertensive. According to the 2017 ACC/AHA guideline, individuals who have an SBP greater than or equal to 130 mm Hg or a DBP greater than or equal to 80 mm Hg or take any prescribed drugs to control blood pressure were categorized as hypertensive. The category of prehypertension was changed to elevated blood pressure in the 2017 ACC/AHA guideline.^1^ eTable 1 in the Supplement shows definitions, categories, and ascertainment methods of all variables used to estimate the prevalence in this study.

### Statistical Analysis

All variables were first investigated in univariate analyses before estimating the prevalence of hypertension. The normality of the continuous variables was assessed, and variables with a skewed distribution were reported with medians and interquartile ranges (IQRs). The prevalence was estimated for both guidelines; we then estimated the absolute differences between the prevalence of hypertension according to the 2 guidelines. All prevalences and differences were reported with 95% confidence intervals according to blood pressure stages and guidelines. The background characteristics to report the estimates were adapted from the WHO-recommended standard reporting format.^12^

In addition, we reported the prevalence for each of the background characteristics of the study participants. We considered the hierarchical structure of the data set to estimate the prevalence. Stata statistical software version 14.0 (StataCorp) was used to analyze data in this study.^13^ We conducted weighted analysis to adjust for the clustered sampling design of the survey.^10^

## Results

A total of 13 519 weighted participants were included in this analysis. The median (IQR) age of the respondents was 38 (26-53) years, and 57.9% (n = 7821) were women (Table 1). Overall, the median (IQR) SBP and DBP were 113 (104-125) and 77 (70-85) mm Hg, respectively. The JNC 7 described 2869 participants (21.2%) as hypertensive, while the 2017 ACC/AHA categorized 5977 people (44.2%) as hypertensive. Most participants (81.4%) reported having their blood pressure measured previously at least once. Of survey participants categorized as hypertensive per the JNC 7 guideline, 40.4% (n = 1160) knew their hypertension status; this proportion was 23.6% (n = 1408) among those classified as hypertensive per the 2017 ACC/AHA guideline. Only 20.4% of those who would have been considered hypertensive per the JNC 7 guideline were taking antihypertensive medications, while this proportion was 9.8% using the 2017 ACC/AHA guideline (n = 584). Of the people who had hypertension according to the JNC 7 and 2017 ACC/AHA guidelines, about 9.7% (n = 280) and 7.2% (n = 431) had a controlled blood pressure level, respectively. The median (IQR) body mass index (BMI) (calculated as weight in kilograms divided by height in meters squared) of those classified as hypertensive per the JNC 7 guideline (23.1 [20.4-26.3]) was slightly higher compared with those classified as hypertensive per the 2017 ACC/AHA guideline (22.6 [20.2-25.7]) and all respondents (21.4 [19.3-24.2]). Of all survey respondents, 41.0% (n = 5546) had no formal education, regardless of hypertension status. The proportion of urban residents was more than 60% among the overall population as well as among those classified as hypertensive under both guidelines. Unweighted characteristics of the study population are available in eTable 2 in the Supplement.

**Table 1.** Background Characteristics of the Weighted Survey Participants

Characteristics	All Participants (N = 13 519), No. (%)	Participants With Hypertension per JNC 7 (n = 2869), No. (%)	Participants With Hypertension per 2017 ACC/AHA (n = 5977), No. (%)
SBP, median (IQR), mm Hg	113 (104-125)	141 (129-154)	126 (118-140)
DBP, median (IQR), mm Hg	77 (70-85)	93 (89-99)	86 (82-92)
Ever measured blood pressure	10 997 (81.4)	2480 (86.5)	5038 (84.3)
Know hypertension status	1678 (12.4)	1160 (40.4)	1408 (23.6)
Taking antihypertensive medication	584 (4.3)	584 (20.4)	584 (9.8)
Controlled blood pressure level	NA	280 (9.7)	431 (7.2)
Age, y			
Median (IQR)	38.0 (26.0-53.0)	51.0 (39.0-63.0)	44.0 (33.0-57.0)
18-29	4383 (32.4)	266 (9.3)	1079 (18.1)
30-49	5024 (37.2)	1089 (38.0)	2479 (41.5)
50-69	3209 (23.7)	1111 (38.7)	1865 (31.2)
≥70	903 (6.7)	403 (14.0)	553 (9.3)
Sex			
Male	5697 (42.1)	1453 (50.6)	2897 (48.5)
Female	7821 (57.9)	1416 (49.4)	3080 (51.5)
BMI^a^			
Median (IQR)	21.4 (19.3-24.2)	23.1 (20.4-26.3)	22.6 (20.2-25.7)
<18.5	2240 (16.8)	311 (11.1)	682 (11.6)
18.5-24.9	8242 (61.7)	1473 (52.5)	3359 (57.1)
25-29.9	2324 (17.4)	785 (28.0)	1444 (24.6)
≥30	549 (4.1)	236 (8.4)	393 (6.7)
Education			
No formal education	5546 (41.0)	1371 (47.8)	2631 (44.0)
Primary	2324 (17.2)	526 (18.3)	1073 (18.0)
Secondary	3695 (27.3)	659 (23.0)	1521 (25.5)
College or above	1951 (14.4)	311 (10.8)	749 (12.5)
Household wealth status			
Poorest	2408 (17.8)	456 (15.9)	1053 (17.6)
Poorer	2617 (19.4)	558 (19.5)	1175 (19.7)
Middle	2699 (20.0)	483 (16.8)	1090 (18.2)
Richer	2945 (21.8)	557 (19.4)	1206 (20.2)
Richest	2850 (21.1)	814 (28.4)	1451 (24.3)
Place of residence			
Urban	8274 (61.2)	1852 (64.5)	3740 (62.6)
Rural	5244 (38.8)	1017 (35.5)	2237 (37.4)
Ecological zone			
Mountain	860 (6.4)	157 (5.5)	354 (5.9)
Hill	5964 (44.1)	1449 (50.5)	2906 (48.6)
Terai	6695 (49.5)	1263 (44.0)	2716 (45.4)
Province			
1	2392 (17.7)	488 (17.0)	1017 (17.0)
2	2770 (20.5)	450 (15.7)	977 (16.4)
3	2976 (22.0)	740 (25.8)	1446 (24.2)
4	1392 (10.3)	400 (13.9)	772 (12.9)
5	2197 (16.3)	515 (18.0)	1092 (18.3)
6	677 (5.0)	109 (3.8)	265 (4.4)
7	1113 (8.2)	166 (5.8)	406 (6.8)

Abbreviations: ACC/AHA, 2017 American College of Cardiology/American Heart Association Guideline for the Prevention, Detection, Evaluation, and Management of High Blood Pressure in Adults; BMI, body mass index; DBP, diastolic blood pressure; IQR, interquartile range; JNC 7, Seventh Report of the Joint National Committee on Prevention, Detection, Evaluation, and Treatment of High Blood Pressure; NA, not applicable; SBP, systolic blood pressure.

^a^ Calculated as weight in kilograms divided by height in meters squared.

Table 2 summarizes the prevalence (with 95% confidence interval) of hypertension among men and women according to the 2 guidelines, along with the absolute difference in prevalence comparing the previous guidelines with the new guideline. The crude prevalence of hypertension was 21.2% (95% CI, 20.5%-21.9%) according to the JNC 7 guideline, compared with 44.2% (95% CI, 43.4%-45.0%) by the 2017 ACC/AHA guideline. More than half of the male respondents had hypertension according to the new classification (50.8% [95% CI, 49.5%-52.2%]), compared with 39.4% (95% CI, 38.3%-40.5%) of the female respondents. The prevalence for hypertension according to JNC 7 was 25.5% (95% CI, 24.4%-26.6%) among men and 18.1% (95% CI, 17.3%-19.0%) among women. Under the new guideline, the overall prevalence of hypertension was 23.0% (95% CI, 22.3%-23.7%) higher because of an equal reduction in the prevalence of prehypertension. Male respondents had a higher absolute increase in prevalence than their female counterparts (25.3% [95% CI, 24.2%-26.5%] and 21.3% [95% CI, 20.4%-22.2%], respectively). A similar percentage increase was observed for stage 1 and stage 2 hypertension for both sexes. The overall absolute increases for the prevalence of stage 1 and stage 2 hypertension were 10.6% (95% CI, 8.4%-12.8%) and 12.4% (95% CI, 11.8%-12.9%), respectively.

**Table 2.** Weighted Prevalence and Absolute Changes in Prevalence According to JNC 7 and 2017 ACC/AHA Guideline

Blood Pressure	Prevalence per JNC 7, % (95% CI)	Prevalence per 2017 ACC/AHA, % (95% CI)	Absolute Difference, % (95% CI)
Men			
Normal	43.8 (42.5-45.1)	43.8 (42.5-45.1)	0
Prehypertension or elevated blood pressure	30.7 (29.5-31.9)	5.4 (4.8-6.0)	−25.3 (−24.2 to −26.5)
Stage 1 hypertension	17.3 (16.4-18.4)	27.8 (26.7-29.0)	10.5 (7.2-13.9)
Stage 2 hypertension	8.1 (7.5-8.9)	23.0 (21.9-24.1)	14.9 (13.9-15.8)
Hypertension (stage 1 plus stage 2)	25.5 (24.4-26.6)	50.8 (49.5-52.2)	25.3 (24.2-26.5)
Women			
Normal	57.7 (56.6-58.8)	57.7 (56.6-58.8)	0
Prehypertension or elevated blood pressure	24.1 (23.2-25.1)	2.9 (2.5-3.3)	−21.3 (−20.4 to −22.2)
Stage 1 hypertension	12.6 (11.9-13.4)	23.4 (22.4-24.3)	10.8 (7.7-13.6)
Stage 2 hypertension	5.5 (5.0-6.0)	16.0 (15.3-16.9)	10.5 (9.9-11.2)
Hypertension (stage 1 plus stage 2)	18.1 (17.3-19.0)	39.4 (38.3-40.5)	21.3 (20.4-22.2)
Overall			
Normal	51.9 (51.0-52.7)	51.9 (51.0-52.7)	0
Prehypertension or elevated blood pressure	26.9 (26.2-27.7)	3.9 (3.6-4.3)	−23.0 (−22.3 to −23.7)
Stage 1 hypertension	14.6 (14.0-15.2)	25.2 (24.5-26.0)	10.6 (8.4-12.8)
Stage 2 hypertension	6.6 (6.2-7.0)	19.0 (18.3-19.6)	12.4 (11.8-12.9)
Hypertension (stage 1 plus stage 2)	21.2 (20.5-21.9)	44.2 (43.4-45.0)	23.0 (22.3-23.7)

Abbreviations: ACC/AHA, 2017 American College of Cardiology/American Heart Association Guideline for the Prevention, Detection, Evaluation, and Management of High Blood Pressure in Adults; JNC 7, Seventh Report of the Joint National Committee on Prevention, Detection, Evaluation, and Treatment of High Blood Pressure.

Among categories of individuals with hypertension according to the 2017 ACC/AHA guideline (Table 3), the highest rate was observed among people with a BMI of 30 or greater (71.6% [95% CI, 67.7%-75.3%]), followed by those with a BMI of 25 to 29.9 (62.1% [95% CI, 60.1%-64.1%]). Among different age groups, prevalence was highest among individuals aged 70 years or older (61.3% [95% CI, 58.1%-64.4%]), those aged 50 to 69 years (58.1% [95% CI, 56.4%-59.8%]), and those aged 30 to 49 years (49.3% [95% CI, 47.9%-50.7%]). Individuals in the richest household wealth quintile had higher rates (50.9% [95% CI, 49.1%-52.8%]), as did those living in a hill area (48.7% [95% CI, 47.5%-50.0%]), Province 4 (55.5% [95% CI, 52.8%-58.1%]), Province 5 (49.7% [95% CI, 47.6%-51.8%]), and Province 3 (48.6% [95% CI, 46.8%-50.4%]). None of the subgroups had more than 50% prevalence according to the JNC 7 guideline, with the highest prevalence among people aged 70 years or older (44.6% [95% CI, 41.4%-47.9%]) and those with a BMI of 30 or greater (43.0% [95% CI, 38.9%-47.2%]). Among those who were not hypertensive per the JNC 7 guideline, the new 2017 ACC/AHA guideline was associated with more than a 20% increase in newly identified hypertensive people in most of the categories.

**Table 3.** Weighted Prevalence of Hypertension in Both Sexes According to Selected Demographic Characteristics

Demographic Characteristics	Prevalence of Hypertension per JNC 7, % (95% CI)	Prevalence of Hypertension per 2017 ACC/AHA, % (95% CI)	Difference, % (95% CI)
Age, y			
18-29	6.1 (5.4-6.8)	24.6 (23.4-25.9)	18.5 (17.4-19.7)
30-49	21.7 (20.6-22.8)	49.3 (47.9-50.7)	27.6 (26.4-28.9)
50-69	34.6 (33.0-36.3)	58.1 (56.4-59.8)	23.5 (22.1-25.0)
≥70	44.6 (41.4-47.9)	61.3 (58.1-64.4)	16.7 (14.4-19.2)
BMI^a^			
<18.5	13.9 (12.5-15.4)	30.4 (28.6-32.4)	16.5 (15.1-18.2)
18.5-24.9	17.9 (17.1-18.7)	40.8 (39.7-41.8)	22.9 (22.0-23.8)
25-29.9	33.7 (31.9-35.7)	62.1 (60.1-64.1)	28.4 (26.5-30.2)
≥30	43.0 (38.9-47.2)	71.6 (67.7-75.3)	28.6 (25.0-32.6)
Education			
No formal education	24.7 (23.6-25.9)	47.4 (46.1-48.7)	22.7 (21.6-23.8)
Primary	22.6 (21.0-24.4)	46.2 (44.2-48.2)	23.6 (21.9-25.3)
Secondary	17.8 (16.6-19.1)	41.2 (39.6-42.8)	23.4 (22.0-24.7)
College or above	15.9 (14.4-17.6)	38.4 (36.2-40.6)	22.5 (20.6-24.3)
Household wealth status			
Poorest	18.9 (17.4-20.6)	43.7 (41.8-45.7)	24.8 (23.1-26.6)
Poorer	21.4 (19.8-23.0)	44.9 (43.0-46.8)	23.5 (21.9-25.2)
Middle	17.9 (16.5-19.4)	40.4 (38.6-42.3)	22.5 (21.0-24.1)
Richer	18.9 (17.5-20.4)	41.0 (39.2-42.8)	22.1 (20.6-23.6)
Richest	28.5 (26.9-30.2)	50.9 (49.1-52.8)	22.4 (20.9-23.9)
Place of residence			
Urban	22.4 (21.5-23.3)	45.2 (44.1-46.3)	22.8 (21.9-23.7)
Rural	19.4 (18.4-20.5)	42.7 (41.3-44.0)	23.3 (22.1-24.4)
Ecological zone			
Mountain	18.3 (15.8-21.0)	41.2 (38.0-44.5)	22.9 (20.2-25.9)
Hill	24.3 (23.2-25.4)	48.7 (47.5-50.0)	24.4 (23.4-25.5)
Terai	18.9 (17.9-19.8)	40.6 (39.4-40.7)	21.7 (20.7-22.7)
Province			
1	20.4 (18.8-22.1)	42.5 (40.6-44.5)	22.1 (20.5-23.8)
2	16.2 (14.9-17.7)	35.3 (33.5-37.1)	19.1 (17.6-20.5)
3	24.9 (23.3-26.4)	48.6 (46.8-50.4)	23.7 (22.2-25.3)
4	28.8 (26.4-31.2)	55.5 (52.8-58.1)	26.7 (24.4-29.1)
5	23.4 (21.7-23.3)	49.7 (47.6-51.8)	26.3 (24.4-28.1)
6	16.2 (13.6-19.2)	39.2 (35.6-42.9)	23.0 (20.0-26.3)
7	14.9 (12.9-17.1)	36.5 (33.7-39.4)	21.6 (19.3-24.1)

Abbreviations: ACC/AHA, 2017 American College of Cardiology/American Heart Association Guideline for the Prevention, Detection, Evaluation, and Management of High Blood Pressure in Adults; BMI, body mass index; JNC 7, Seventh Report of the Joint National Committee on Prevention, Detection, Evaluation, and Treatment of High Blood Pressure.

^a^ Calculated as weight in kilograms divided by height in meters squared.

## Discussion

We investigated the change in estimated prevalence of hypertension in Nepal according to the lower blood pressure threshold recommended in the 2017 ACC/AHA guideline. Under the new guideline, 44.2% of adults in Nepal are now considered to have hypertension. In addition, regardless of background characteristics of the survey participants, these findings reclassified more than one-fifth of adults as hypertensive who were categorized as prehypertensive according to the JNC 7 guideline.

The 2017 ACC/AHA guideline recommended a lower blood pressure threshold for the diagnosis of hypertension based on medical evidence indicating that even a small increase in blood pressure increases risks of morbidity and mortality.^1^ Application of the new guideline should have a significant impact on hypertension prevention and management in countries like Nepal, where only one-fifth of the adult population who had hypertension according to previous guidelines (ie, the JNC 7 or 1999 WHO-ISH) were taking blood pressure–lowering drugs. In addition to our study, other studies that investigated the awareness and control of blood pressure among adults in Nepal found an overall lower level of control and awareness of hypertension in this country.^14,15^ Furthermore, like many other developing countries, Nepal is facing a double burden of infectious and noncommunicable diseases that warrants national awareness and control programs.^16,17^

Despite the similar absolute difference for prevalence of hypertension regardless of background characteristics, the 2017 ACC/AHA prevalence was higher depending on some background characteristics, such as age, BMI, and household wealth status; the same groups also had higher prevalence using the JNC 7 threshold. The determinants of hypertension are beyond the scope of this discussion; however, studies that investigated risk factors for hypertension in Nepal found a higher likelihood of hypertension among individuals who were older, had a higher BMI, and belonged to the highest quintile of household wealth.^18,19,20,21^ These high-risk groups require more awareness and control of hypertension to minimize complications or negative consequences associated with hypertension.^1^

The 2017 ACC/AHA guideline recommends treating stage 1 hypertension with lifestyle measures plus antihypertensive drugs for secondary prevention of clinical cardiovascular disease or for primary prevention of cardiovascular disease if the estimated 10-year risk of atherosclerotic cardiovascular disease is 10% or higher. Additionally, persons with stage 2 hypertension and an estimated 10-year risk of atherosclerotic cardiovascular disease greater than 10% are recommended to take antihypertensive drugs along with lifestyle measures. Patients with stage 1 hypertension who have a 10-year risk of atherosclerotic cardiovascular disease greater than 10% or elevated blood pressure would require lifestyle modification.^1^ Our findings are also essential in the context that at least half of the adults in Nepal (those with either hypertension or elevated blood pressure) should follow active lifestyles and healthy dietary habits.^1^ Public health programs should encourage active lifestyles and healthy diets among all people in this country, not just those with hypertension. We were unable to estimate the proportion of patients who could require antihypertensive medication because of limitations of the NDHS 2016 data set. This estimation is required to fully understand the burden of hypertension in Nepal.

Of the few studies of which we are aware that examined the prevalence of hypertension according to the latest guideline, only Muntner et al^6^ worked with a similar age group (ie, ≥20 years). They also found a similar absolute change in prevalence of hypertension after applying the new 2017 ACC/AHA guideline. This finding is important not only because Nepal and the United States are socioeconomically or demographically different, but also because each population had a substantial difference (10.4%) in the prevalence of hypertension according to the JNC 7 guideline for this age group.^6,10^ Using the JNC 7 guideline, hypertension prevalence was 21.2% in Nepal and 31.6% in the United States. However, the 2017 ACC/AHA classified 44.2% and 45.6% of people as hypertensive in Nepal and the United States, respectively; the new estimates are very close to each other (1.4%).^6^ Given the similar prevalence of hypertension in many other countries according to the JNC 7 guideline’s hypertension definition,^22,23^ the prevalence of hypertension in other countries could be similar according to the 2017 ACC/AHA guideline’s thresholds. However, it is necessary to estimate both the prevalence of hypertension and the proportion requiring pharmacologic treatment for hypertension according to the 2017 ACC/AHA guideline in other countries to estimate the overall global burden of hypertension. In addition, evaluating the degree of awareness and control of blood pressure levels in Nepal and other countries according to the 2017 ACC/AHA guideline may be helpful in understanding the future research requirements needed to overcome this massive public health challenge.

The strengths of this study include generalizability of the findings for Nepal, as this survey covered both urban and rural areas of all provinces in this country,^10^ and also the use of appropriate statistical methods to estimate the weighted prevalence of hypertension from the study sample.

### Limitations

The limitations of the current study also warrant discussion. The survey data set was cross-sectional, and blood pressure of the participants was measured 3 times in a single day. Both guidelines recommend longitudinal measurement of blood pressure levels to diagnose hypertension. Furthermore, this survey used an automated device, while both guidelines recommend recording blood pressure with a sphygmomanometer.^1,6,10^ Although the survey staff were highly trained, their efficacy or skill level may cause some misclassification.^6,10,24^

## Conclusions

The results of our study indicate that a significant proportion of the adults in Nepal may have hypertension according to the latest guideline. Considering the morbidity and mortality associated with hypertension, this condition is a major public health challenge for Nepal and other countries with similar sociodemographic characteristics. Our results signify the importance of implementing more awareness programs to control hypertension as well as minimizing complications associated with hypertension. In addition, similar research in other countries could be helpful to estimate country-specific burdens of hypertension.

## Notes

Supplement. **eTable 1.** Description of Study Variables **eTable 2.** Background Characteristics of the Unweighted Survey Participants
