# Supplementary material for: Estimated Change in Prevalence of Hypertension in Nepal Following Application of the 2017 ACC/AHA Guideline
Source: JAMA Netw Open. 2018 Jul 13;1(3):e180606. doi: 10.1001/jamanetworkopen.2018.0606 (PMC6324293; doi:10.1001/jamanetworkopen.2018.0606)
Supplement: Supplement. — eTable 1. Description of Study Variables eTable 2. Background Characteristics of the Unweighted Survey Participants [file jamanetwopen-1-e180606-s001.pdf]

Supplementary Online Content

Kibria GMA, Swasey K, KC A, et al. Estimated change in prevalence of hypertension in Nepal following application of the 2017 ACC/AHA guideline. *JAMA Netw Open*. 2018;1(3):e180606. doi:10.1001/jamanetworkopen.2018.0606

eTable 1. Description of Study Variables

eTable 2. Background Characteristics of the Unweighted Survey Participants

This supplementary material has been provided by the authors to give readers additional information about their work.

© 2018 Kibria GMA et al. *JAMA Network Open.*

eTable 1 : Description of study variables

Study variables	Ascertainment	Definitions	Categories
Crude Hypertension	The average of last two measurements.	JNC7: An SBP ≥140 mm Hg or DBP ≥90 mm Hg or a person is taking any prescribed drugs to control raised blood pressure.<br><br>2017 ACC/AHA: An SBP ≥130 mm Hg or a DBP ≥80 mm Hg or a person is taking any prescribed drugs to control raised blood pressure.	Dichotomous.<br>Yes, no
Prehypertension / Elevated blood preesure*	The average of last two measurements.	Pre-hypertension (JNC7): An SBP 120-139 mm Hg or DBP 80-89 mm Hg and the person is not taking any prescribed drugs to control raised blood pressure.<br><br>Elevated blood pressure (2017 ACC/AHA) : An SBP 120-129 mm Hg and DBP <80 mm Hg and the person is not taking any prescribed drugs to control raised blood pressure.	Dichotomous.<br>Yes, no
Stage-1 Hypertension	The average of last two measurements.	JNC7: An SBP 140-159 mm Hg or DBP 90-99 mm Hg.<br><br>2017 ACC/AHA: An SBP 130-139 mm Hg or DBP 80-89 mm Hg.	Dichotomous.<br>Yes, no
Stage-2 Hypertension	The average of last two measurements.	JNC7: An SBP ≥160 mm Hg or DBP ≥100 mm Hg.<br><br>2017 ACC/AHA: An SBP ≥140 mm Hg or DBP ≥90 mm Hg.	Dichotomous.<br>Yes, no
Age	Verbal report.	Age of the respondents in years	Ordinal.<br>18-29; 30-49; 50-69; ≥70
Sex	Verbal report.	Gender of the respondents.	Dichotomous.<br>Male, female.
Body Mass Index (BMI)	Physical measurement.	BMI of the respondents (kg/m^2). Obtained by dividing weight (in kilograms) with square of the height (in meters).	Ordinal.<br><18.5, 18.5-24.9, 25-29.9, ≥30.
Education	Verbal report.	Education level of the respondents.	Ordinal.<br>No formal education, primary (1-5 years), Secondary (6-10 years), college (≥11 years)

© 2018 Kibria GMA et al. *JAMA Network Open.*

Household wealth status	Principal component analysis.	Composite index of household materials; obtained by principal component analysis.	Ordinal. Poorer, poorer, middle, richer, richest.
Place of residence	Place of interview.	Whether the person is living in a rural or urban area at the time of interview.	Dichotomous. Urban, rural.
Ecological zone	Ecological zone of interview.	Based on climate and landform Nepal is divided into three ecological zones.	Poly-chotomous. Mountain, Hill, Terai
Province	Province of interview.	Province of residence. Province is the largest administrative unit in Nepal.	Poly-chotomous. Provinces 1-7.

SBP: Systolic blood pressure, DBP: Diastolic blood pressure,

© 2018 Kibria GMA et al. *JAMA Network Open.*

eTable 2: Background characteristics of the un-weighted survey participants[1]

Characteristics	Overall (n=13,432)	Among those with hypertension according to guideline	
		JNC7 (n=2,792)	2017 ACC/AHA (n=5,940)
SBP, Median (IQR)	113 (104-125)	141 (129-154)	126 (118-140)
DBP, Median (IQR)	77 (70-85)	93 (89-99)	86 (82-92)
Ever measured blood pressure	10,872 (80.9)	2,402 (86.0)	4,960 (83.5)
Know hypertension status	1,611 (12.0)	1,109 (39.7)	1,348 (22.7)
Taking anti-hypertensive	541 (4.0)	541 (19.4)	541 (9.1)
Controlled pressure level		267 (9.6)	402 (6.8)
Age (years)			
Median (IQR)	38.0 (26.0-53.0)	51.0 (39.0-63.0)	44.0 (33.0-57.0)
18-29	4,360 (32.5)	271 (9.7)	1,117 (18.8)
30-49	4,971 (37.0)	1,041 (37.3)	2,442 (41.1)
50-69	3,215 (23.9)	1,085 (38.9)	1,833 (30.9)
≥70	886 (6.6)	395 (14.2)	548 (9.2)
Sex			
Male	5,571 (41.5)	1,404 (50.3)	2,846 (47.9)
Female	7,861 (58.5)	1,388 (49.7)	3,094 (52.1.5)
Body mass index (kg/m^2)			
Median (IQR)	21.4 (19.3-24.2)	23.1 (20.4-26.3)	22.6 (20.2-25.7)
<18.5	2,218 (16.7)	297 (10.9)	670 (11.5)
18.5-24.9	8,421 (63.5)	1,475 (54.0)	3,436 (58.8)
25-29.9	2,150 (16.2)	745 (27.3)	1,372 (23.5)
≥30	482 (3.6)	213 (7.8)	361 (6.2)
Education			
No formal education	5,576 (41.5)	1,365 (48.9)	2,648 (44.6)
Primary	2,304 (17.2)	517 (18.5)	1,082 (18.2)
Secondary	3,697 (27.5)	627 (22.5)	1,501 (25.3)
College or above	1,852 (13.8)	281 (10.1)	706 (11.9)
Household wealth status			
Poorest	2,896 (21.6)	525 (18.8)	1,235 (20.8)
Poorer	2,747 (20.5)	581 (20.8)	1,222 (20.6)
Middle	2,673 (19.9)	491 (17.6)	1,114 (18.8)
Richer	2,745 (20.4)	551 (19.7)	1,166 (19.6)
Richest	2,371 (17.7)	644 (23.1)	1,203 (20.23)
Place of residence			
Urban	8,484 (63.2)	1,838 (65.8)	3,807 (64.1)
Rural	4,948 (36.8)	954 (34.2)	2,133 (35.9)
Ecological zone			
Mountain	999 (7.4)	166 (6.0)	382 (6.4)
Hill	6,123 (45.6)	1,439 (51.5)	2,974 (50.1)
Terai	6,310 (47.0)	1,187 (42.5)	2,584 (43.5)
Province			
Province 1	2,021 (15.0)	410 (14.7)	852 (14.3)
Province 2	2,241 (16.7)	378 (13.5)	821 (13.8)

© 2018 Kibria GMA et al. *JAMA Network Open.*

Province 3	1,928 (14.4)	481 (17.2)	979 (16.5)
Province 4	1,800 (13.4)	511 (18.3)	993 (16.7)
Province 5	2,008 (15.0)	469 (16.8)	990 (16.7)
Province 6	1,632 (12.2)	278 (10.0)	654 (11.0)
Province 7	1,802 (13.4)	265 (9.5)	651 (11.0)

JNC: Joint National Committee; ACC/AHA: American College of Cardiology/American Heart Association; SBP: Systolic blood pressure; DBP: Diastolic blood pressure; IQR: inter-quartile range;
1.All numbers are presented as n(column percentage) unless indicated otherwise

© 2018 Kibria GMA et al. *JAMA Network Open.*
